# Assessing the magnitude of burnout among emergency nurses in Portugal

**DOI:** 10.3389/fpubh.2025.1699355

**Published:** 2025-11-13

**Authors:** Júlio Belo Fernandes, Ricardo Antunes, Maria Ivanel, Mariana Lucas, Ricardo Oliveira, Wilson Bico, Aida Simões, Diana Vareta, Catarina Bernardes, Célia Vaz, Steven Hall, Sónia Fernandes, Cidália Castro

**Affiliations:** 1 Egas Moniz Center for Interdisciplinary Research (CiiEM), Egas Moniz School of Health and Science, Almada, Portugal; 2 Nurs^*^ Lab, Almada, Portugal; 3 Faculty of Nursing, University of Alberta, Edmonton, AB, Canada

**Keywords:** burnout, professional, nurses, emergency nursing, emergency service, hospital

## Abstract

**Background:**

Burnout is a health condition associated with chronic work-related stress. Nurses working in hospital emergency rooms are particularly susceptible to experiencing burnout. It is crucial to understand the phenomena of burnout among emergency room nurses, particularly in light of the COVID-19 pandemic that healthcare professionals have confronted. This study aims to evaluate burnout among nurses working in emergency rooms while examining the relationship between burnout and sociodemographic and occupational variables.

**Methods:**

This descriptive, observational, and cross-sectional study utilized a web-based survey administered to 112 nurses from eight hospital emergency rooms in the Lisbon metropolitan area between November 2022 and February 2023. Burnout was measured using the Portuguese version of the Maslach Burnout Inventory (MBI), which assessed three subscales: Emotional Exhaustion (EE), Depersonalization (DP), and Personal Accomplishment (PA). The relationship between burnout and sociodemographic and professional characteristics of nurses in emergency rooms was analyzed using Chi-square tests and one-way ANOVA.

**Results:**

The prevalence of burnout was 56.6%, with 27.4% experiencing severe burnout. The three subscales of the MBI showed high prevalence rates: 49.1% for EE, 44.6% for DP, and 38.4% for low PA. Severe burnout and high EE were associated with younger age, being single, not having children, having less professional experience, less graduate training, and having more precarious employment contracts.

**Conclusions:**

Three years after the onset of the COVID-19 pandemic, the results highlight the ongoing critical situation arising from the cumulative effects of the crisis on the Portuguese healthcare system, leading to high rates of burnout and EE among emergency room nurses.

## 1 Introduction

Since the 1970s, the conceptualization of burnout has evolved. Initially, Freudenberger (1) defined it as physical and mental exhaustion resulting from constant emotional and interpersonal tensions. Maslach and Jackson (2) further characterized burnout as an individual response to excessive and prolonged stress, specifically in the workplace. In 2019, burnout gained recognition at the 72nd World Health Assembly and was included in the 11th Revision of the International Classification of Diseases (ICD-11) (3). According to the ICD-11, burnout is described as a syndrome resulting from chronic workplace stress with which the individual cannot cope (4). Although burnout is not classified as a disease but as an occupational phenomenon, it can lead to decreased mental wellness and commonly leads to individuals seeking health services (4). Studies and meta-analyses reveal that health professionals are at greater risk for burnout in the workplace (5, 6). In this context, and compared to other health professions, nurses show more symptoms of post-traumatic stress (7), depression (8) and have a higher risk of developing burnout (9, 10). Research in this field has identified several contributing factors, including studies involving nursing students (11, 12). Nursing care practices include direct interaction with patients in different stages of illnesses (13, 14). Nurses within the hospital's organizational hierarchy often experience less autonomy than their medical professionals (15, 16). The chronic shortage of nurses in health services (5, 16) translates into increased workloads, frequent extra shifts, and a subsequent increase in sick leave (17). These challenges, in addition to the prolonged exposure to stress and direct contact with patients' and families' suffering, contribute to a higher risk of developing mental health problems among nurses (13, 18).

Emergency rooms can intensify these risk factors (5, 19), as they are characterized by an accelerated pace, uncertainty, and indetermination, where nurses are continuously exposed to traumatic and distressing events such as severe injuries, death, and suicide (20). Moreover, nurses in these settings often suffer verbal and physical aggression from patients and their families (10, 21). Furthermore, the demand for emergency rooms has risen continuously. In Portugal, in the last 25 years, there has been an increase of 30% in the number of users assisted in Portugal's emergency rooms (22). Similarly, in North America, Canadian emergency rooms saw 15.5 million unscheduled visits in 2023–2024—an almost 3% increase from the prior year (23). Consequently, nurses working in emergency rooms tend to experience higher burnout rates compared to their counterparts in other hospital services (8, 19, 24). These challenging circumstances have been further exacerbated by the COVID-19 pandemic, which led to increased demand for health services, heightened stress levels, excessive workloads, and an escalation of burnout rates among emergency room professionals (25, 26), including nurses (24, 27).

In light of these circumstances, Maslach's et al. offers a valuable tool for assessing burnout, utilizing the Maslach Burnout Inventory (MBI) as an assessment scale widely applied across various work domains (28). The MBI consists of three dimensions: emotional exhaustion (EE), which encompasses physical and mental exhaustion and the feeling of reaching one's limits; depersonalization (DP), involving a shift in interpersonal feelings, leading to a more distant and impersonal attitude toward others' suffering; and decreased personal accomplishment (PA), reflecting the negative impact of external factors on an individual's wellbeing and professional relationships, often manifested as reduced feelings of competence and performance (29). This study aims to assess burnout among emergency room nurses working while also examining the relationship between burnout and sociodemographic and occupational variables.

## 2 Methods

### 2.1 Study design

This study corresponds to the first phase of a broader research project developed in three stages, aimed at validating an intervention program for preventing burnout among emergency room nurses. The initial phase, presented in this paper, focuses on conducting a situational diagnosis to understand the prevalence and severity of burnout in this population and identify the sociodemographic and professional factors associated with increased risk. The findings of this diagnostic phase are critical to confirming the need for targeted interventions and to inform the design and tailoring of the subsequent intervention strategies that will be developed and tested in the following phases of the project. For this phase, we conducted a descriptive, observational, cross-sectional study.

### 2.2 Setting and sample

A convenience sample was drawn from nurses working in the emergency rooms of public hospitals in the Lisbon metropolitan area. Ethics approval was obtained by the Egas Moniz CRL Ethical Review Committee (ID: 984). We contacted the nursing heads of the emergency rooms, explained the purpose of the study, and sought their collaboration in recruiting participants. A non-probabilistic convenience sample was used where all nurses working in emergency services were considered for this study. Through social networks, the nurses were invited to participate by accessing a link to the questionnaire.

### 2.3 Data collection

Data were collected in an online survey between November 2022 and February 2023. Before completing the survey, each nurse participant received a detailed explanation of the study's procedures and objectives and provided informed consent. Participation in the research was entirely voluntary and anonymous. Before the official survey, a pre-test was administered, and the final online survey was conducted. On average, it took participants 15 minutes to complete the questionnaire.

### 2.4 Measures/instruments

Burnout was assessed using the MBI, one of the most widely used instruments in nursing research (28). The MBI comprises of three different versions tailored to specific professional domains. In this study, we employed the Portuguese-validated version of the MBI-HSS (Human Services Survey) (30), which has also been utilized in previous research (31).

The MBI-HSS self-assessment instrument comprises 22 questions rated on a Likert-type scale that ranges from 0 (never) to 6 (daily). It is divided into three dimensions, represented by separate subscales, which examine the extent of burnout: emotional exhaustion (EE), depersonalization (DP), and lack of personal achievement (PA) (29). Burnout levels can be categorized as low, moderate, or high. According to Maslach et al. (29), score cut points for each burnout subscale have been empirically determined. The score for each subscale is calculated by summing the values of all the responses. For instance, a high level of burnout is indicated by a sum score ≥27 in EE, a sum score ≥13 in DP, and a sum score ≤ 31 in PA (29). Consistent with the approach adopted by Elmore et al. (32), overall burnout was defined as scoring above the threshold on at least one of the three subscales, whereas severe burnout was defined as scoring above the thresholds on all three subscales. For the PA dimension, lower scores indicate higher levels of burnout. (29, 32).

### 2.5 Statistical analyses

Data were organized and classified using statistical treatment based on the IBM SPSS Statistics^®^ for windows, v. 27.0. IBM Corp. Released 2020, Armonk, NY, USA. All variables were analyzed using descriptive statistics. Categorical variables were presented as numbers and percentages, and continuous variables as mean and standard deviation. As appropriate, statistical significance was tested for associations between variables using Chi-square test and ANOVA. Data normality was assessed using the Kolmogorov–Smirnov test and graphical inspection, while homogeneity of variances was evaluated with Levene's test. *P*-values less than 0.05 were considered statistically significant. In the event that distributions were non-Gaussian, appropriate non-parametric methods (e.g., Mann–Whitney U, Wilcoxon signed-rank, Kruskal–Wallis) or data transformations (e.g., logarithmic, square-root) would be applied.

## 3 Results

### 3.1 Sample characteristics

During the designated survey period, we received a total of 122 responses. After organizing the data and applying criteria for missing or incomplete responses, non-responses, and compliance with inclusion criteria, we obtained a final sample comprising 112 nurses from the emergency rooms in eight of the initially selected hospitals. As shown in Table 1, in this sample, the majority of respondents were female (78.6%), under 40 years old (69.6%), with a mean age of 35.3 years (SD = 9.72).

**Table 1:** Sociodemographic and occupational characteristics of the sample (*n* = 112).

**Variable**	**Group**	***N***	**%**
Sex	Male	24	21.4
	Female	88	78.6
Age	29 ≤	42	37.5
	30–39	36	32.1
	≥40	34	30.4
Relationship status	Single/divorcee/widow	52	46.4
	Married/non-marital partnerships	60	53.6
Have children	Yes	47	42.0
	No	65	58.0
Work experience as a nurse (years)	0–4	40	36.0
	5–9	17	15.3
	10–14	22	19.8
	≥15	32	28.8
Nursing specialization	Yes	39	34.8
	No	73	65.2
Employment contract	Civil servant contract	41	36.6
	Others	71	63.4
Wanted to change profession in the last month	Yes	53	47.7
	No	58	52.3

Regarding family dimensions, approximately half of the participants (53.6%) were in a marital or informal relationship, and 42.0% reported having children. The average nursing experience among participants was 10.5 years (SD = 6.98). Around half of the nurses (51.3%) had been working for at least 9 years, and approximately one-third (28.8%) had nursing experience of over 15 years. About one-third of the nurses (34.8%) had specialized in a specific area of nursing, with medical-surgical nursing being the most common specialization. Regarding employment contracts, 36.6% were employed as civil servants with a contract with the state, while the remaining 63.4% had fixed-term or other less stable contractual arrangements. Finally, around 47.7% of the respondents desired to change their profession in the last month.

### 3.2 MBI assessment findings

In this study, the outcomes of interest were defined as binary variables indicating high EE and DP scores, and a low PA score. Table 2 presents their prevalence: high EE was observed in 49.1% of participants, high DP in 44.6%, and low PA in 38.4%. Overall burnout, defined as scoring above the threshold on at least one of the three subscales, was present in 56.6% of participants, while severe burnout, defined as scoring above the thresholds on all three subscales, was identified in 27.4%. The internal consistency of the MBI subscales was satisfactory. Cronbach's alpha coefficients indicated good internal consistency for the MBI subscales: 0.87 for EE, 0.81 for DP, and 0.76 for PA.

**Table 2:** Prevalence of the 3 dimensions of the MBI, overall burnout and severe burnout (*n* = 112).

**Domain**	***N***	**%**
High Emotional Exhaustion (EE)	55	49.1
High Depersonalization (DP)	50	44.6
Low Personal Achievement (PA)	43	38.4
Overall burnout	64	56.6
Severe burnout	31	27.4

Concerning the results of the three dimensions of the MBI scale, Table 2 displays the prevalence of the most extreme burnout situations in each of the three subscales: high EE was found in 49.1% of participants, high DP in 44.6%, and low PA in 38.4%. When analyzing the combined scores of these three dimensions, the overall prevalence of burnout was 56.6%, indicating a score in the most extreme tertile on at least one of the three MBI subscales. The prevalence of severe burnout, characterized by scores in the most extreme tertile on all three MBI subscales, was 27.4%. In this study, the internal consistency of the MBI subscales was evaluated using Cronbach's alpha coefficients: EE = 0.87, DP = 0.81, and PA = 0.76.

Following Maslach et al. (29) cut-off criteria, burnout levels were initially classified as low, moderate, or high. For greater analytical clarity and to highlight the most severe form, the *medium* and *low* categories were merged, resulting in two final groups: “Severe burnout” and “Medium and low burnout”, as shown in Table 3.

**Table 3:** Associations of burnout and severe burnout with sociodemographic and occupational characteristics (*n* = 112).

**Variable**	**Group**	**Medium low burnout (%)**	**Severe burnout (%)**	***X* ^2^**	***P***
Sex	Male	58.3	41.7	2.871	0.122
	Female	75.9	24.1		
Age	≤ 29	61.9	38.1	5.117	0.077
	30–39	71.4	28.6		
	≥40	85.3	14.7		
Relationship status	Single/divorcee/widow	62.7	37.3	4.078	0.560
	Married/non-marital partnerships	80.0	20.0		
Have children	Yes	87.0	13.0	8.646	0.005
	No	61.5	38.5		
Work experience as a nurse (years)	0–4	61.5	38.5	3.778	0.286
	5–9	70.6	29.4		
	10–14	77.3	22.7		
	≥15	81.3	18.8		
Nursing specialization	Yes	84.6	15.4	4.673	0.045
	No	65.3	34.7		
Employment contract	Civil Servant	82.9	17.1	5.193	0.280
	Others	65.7	34.3		
Wanted to change profession in the last month	Yes	59.6	40.4	7.256	0.010
	No	82.8	17.2		

Among the sociodemographic and occupational variables examined (Table 3), three factors showed statistically significant associations with severe burnout. Nurses with children were less likely to experience severe burnout compared to those without children (13.0% vs. 38.5%; χ^2^ = 8.646, *p* = 0.005), suggesting that parental responsibilities may act as a protective factor. Similarly, specialist nurses reported lower rates of severe burnout than their non-specialist counterparts (15.4% vs. 34.7%; χ^2^ = 4.673, *p* = 0.045), highlighting the potential buffering role of advanced training or professional recognition. In contrast, the strongest risk factor was the intention to leave the profession: nurses who had considered changing careers in the past month exhibited significantly higher frequencies of severe burnout (40.4% vs. 17.2%; χ^2^ = 7.256*, p* = 0.010). These findings indicate that family context, professional specialization, and career dissatisfaction play central roles in shaping vulnerability to burnout.

### 3.3 MBI subscale scores in relation to nurse characteristics

The scores of the three MBI subscales, EE, DP, and PA, were analyzed in articulation with the characteristics of the sample of nurses, using independent samples t-tests and ANOVA (Table 4).

**Table 4:** MBI burnout subscales scores and characteristics of emergency room nurses (*n* = 112).

**Variable**	**Group**	**Emotional exhaustion**			**Depersonalization**			**Personal achievement**		
		***M***	**SD**	***p***	***M***	**SD**	***p***	***M***	**SD**	***p***
Sex	Male	29.6	11.79	0.604	12.4	5.67	0.206	30.0	9.08	0.152
	Female	28.2	11.87		10.8	5.65		32.6	7.21	
Age (years)	≤ 29	31.1	10.02	0.025	11.3	5.26	0.106	31.1	6.60	0.002
	30–39	29.8	11.47		12.4	5.82		29.7	9.07	
	≥40	24.0	13.22		9.6	5.78		35.7	6.01	
Relationship status	Single/divorcee/widow	30.9	10.99	0.042	11.7	5.83	0.303	30.3	7.88	0.023
	Married/non-marital partnerships	26.4	12.19		10.6	5.52		33.6	7.22	
Have children	Yes	23.0	12.27	0.001	9.2	5.33	0.002	34.4	7.59	0.004
	No	32.5	9.79		12.5	4.56		30.3	7.31	
Work experience as a nurse (years)	0–4	30.7	9.39	0.006	11.6	5.50	0.214	30.7	6.61	0.062
	5–9	32.5	9.98		11.9	4.38		31.9	6.82	
	10–14	30.1	10.77		12.4	5.44		30.0	8.19	
	≥ 15	22.5	14.32		9.5	6.31		35.0	8.51	
Nursing specialization	Yes	24.4	12.28	0.007	9.6	5.50	0.044	34.9	6.54	0.004
	No	30.7	11.04		11.9	5.64		30.5	7.85	
Employment contract	Civil servant contract	25.2	13.05	0.029	10.0	5.95	0.105	34.4	7.89	0.004
	Others	30.3	10.75		11.8	5.44		30.7	7.28	
Wanted to change profession in the last month	Yes	31.8	10.11	0.005	12.9	4.85	0.002	29.5	7.11	0.001
	No	25.5	12.65		9.6	5.85		34.3	7.58	

According to established cut-off criteria, burnout is defined by an EE score ≥ 27, a DP score ≥ 13, and a PA score ≤ 31. Regarding sex, no statistically significant differences were found between male and female nurses across the three subscales. However, mean values indicated a trend toward higher EE among men (*M* = 29.6 vs. 28.2) and higher PA among women (*M* = 32.6 vs. 30.0). Both groups exceeded the EE cut-off, indicating high emotional exhaustion regardless of sex. Age was significantly associated with EE (*p* = 0.025) and PA (*p* = 0.002). Nurses aged ≤ 29 years reported the highest EE (*M* = 31.1; SD = 10.02), surpassing the cut-off, whereas those ≥40 years exhibited substantially lower EE (*M* = 24.0; SD = 13.22). Conversely, PA increased with age, with nurses ≥40 years reporting levels well above the cut-off (*M* = 35.7; SD = 6.01), suggesting a protective role of age against burnout. Marital status was also relevant: single, divorced, or widowed nurses reported significantly higher EE (*M* = 30.9; SD = 10.99; *p* = 0.042) and lower PA (*M* = 30.3; SD = 7.88; *p* = 0.023) compared with married or partnered nurses (*M* = 26.4; SD = 12.19 for EE; *M* = 33.6; SD = 7.22 for PA). Importantly, the mean PA of single/divorced/widowed nurses was at the burnout threshold ( ≤ 31). Parental status demonstrated a consistent protective effect across all dimensions. Nurses with children reported significantly lower EE (*M* = 23.0; SD = 12.27; *p* = 0.001) and DP (*M* = 9.2; SD = 5.33; *p* = 0.002), alongside higher PA (*M* = 34.4; SD = 7.59; *p* = 0.004). In contrast, childless nurses exceeded the EE cut-off (*M* = 32.5; SD = 9.79) and had a mean PA close to the burnout threshold (*M* = 30.3; SD = 7.31). Length of service was inversely associated with EE (*p* = 0.006). Nurses with ≥15 years of professional experience showed the lowest EE (*M* = 22.5; SD = 14.32) and early-career nurses (0–9 years) reported scores well above the EE cut-off suggesting greater vulnerability. Specialization was significantly associated with reduced burnout. Specialized nurses presented lower EE (*M* = 24.4; SD = 12.28; *p* = 0.007) and DP (*M* = 9.6; SD = 5.50 *p* = 0.044), and higher PA (*M* = 34.9; SD = 6.54; *p* = 0.004), suggesting advanced training functions as a protective factor. Employment contract type also played a role. Civil servant nurses reported lower EE (*M* = 25.2; SD = 13.05; *p* = 0.029) and higher PA (*M* = 34.4; SD = 7.89; *p* = 0.004) compared with those under other contracts, indicating that job security may buffer against burnout. Finally, nurses who had considered leaving the profession in the past month exhibited significantly higher EE (*M* = 31.8; SD = 10.11; *p* = 0.005) and DP (*M* = 12.9; SD = 4.85; *p* = 0.002), coupled with lower PA (*M* = 29.5; SD = 7.11; *p* = 0.001). Their mean PA fell below the burnout threshold, identifying this subgroup as the most vulnerable to burnout.

## 4 Discussion

Studies consistently demonstrate that adverse work environments in nursing are associated with burnout, which has severe consequences for both staff and patients (28). Our study's findings, in line with other research (24, 27), confirm that emergency rooms are high-risk contexts for the development of burnout among nurses. Analyzing the levels of burnout and its dimensions, including emotional exhaustion, depersonalization, and personal fulfillment, revealed that age and professional experience of nurses are factors that differentiate the degrees of burnout and its dimensions. The sample's sociodemographic characterization revealed a polarization in age and professional experience among the nurses, with one-third having less than 5 years of experience and another third having more than 15 years of experience. Similar to other studies (33), younger nurses with less professional experience reported higher levels of emotional exhaustion and severe burnout. This highlights the importance of integrating new nurses into the workforce (34, 35), starting from their training period to prepare them for the labor market and reduce burnout among future nurses (11, 12).

Our study found that age and professional experience were the dimensions with the most significant capacity and differentiation in terms of burnout levels, reflecting the stability of work and employment relationships. Job stability, particularly being employed in the civil service, is associated with career progression in nursing within the Portuguese National Health Service and is linked to educational investments in postgraduate studies and nursing specialization. As seen in other studies, factors related to labor relations and organizational support (36), as well as employment status, were relevant in distinguishing burnout levels (28). In our study, nurses with more stable contracts, such as those in the civil service, with integrated nursing specialties and longer tenures, exhibited lower burnout levels. The family dimension also highlighted the importance of social and affective bonds.

Consistent with other investigations that focused on social support (8), our study found that being in a relationship (37) and having children (38) were protective factors against severe burnout. Additionally, our results showed that high levels of burnout were associated with a desire to change professions, as highlighted by Maslach et al. (29). Importantly, our study revealed high prevalence rates of burnout, with half of the nurses in emergency rooms experiencing burnout, and nearly a third experiencing severe burnout. These rates are higher compared to other studies (8, 13, 17, 24, 36). Among the three subscales of the MBI, EE stood out, as it closely aligns with the classic concept of stress according to Maslach et al. (29), and it was the factor that contributed the most to increased severe burnout.

These results were surprising, considering that the most severe phase of the COVID-19 pandemic had already passed. Enhancing health center environments is essential to mitigate staff burnout and sustain high-quality patient care, particularly under epidemic conditions. The elevated levels of mental exhaustion observed among emergency department physicians and nurses underscore the urgent need for targeted environmental improvements within health services (39). Nevertheless, studies consistently show that the level of burnout among nurses increased during different waves of the COVID-19 pandemic, characterized by high infection and mortality rates (25–27). In this sense, it was expected that after the period of increased pandemic severity, accompanied by a significant decrease in COVID-19 mortality (38), and a return to social normality, burnout rates would be lower. However, our findings deviated from this expectation, necessitating reflection to understand the underlying context.

During the pandemic, healthcare was significantly affected by confinement and access restrictions (40). The number of scheduled surgeries in 2020 decreased by over 20%, and more than a third of Portuguese people (34%) reported unmet healthcare needs during the first 12 months of the pandemic, surpassing the European Union average of 21% (41).

At the end of 2022 and the beginning of 2023, when our investigation took place, the most severe phase of the pandemic had already passed. In Portugal, this period was characterized by a favorable scenario with significant drops in infection levels and a consequent decrease in COVID-19 mortality (38, 42). However, epidemiological reports from the Portuguese Ministry of Health indicated adverse effects of the pandemic on the healthcare system as a whole, particularly the exacerbation of other diseases, especially chronic conditions (42). Among these effects was the increase in the general mortality rate and the severity of situations in more advanced stages of chronic pathologies that arrived at emergency rooms (43). Between November 2022 and January 2023, mortality rates were higher than expected in the Lisbon region, especially among individuals aged 75 years or older, indicating an increased risk of death compared to pre-pandemic years (43).

Although the most severe phase of the pandemic had passed, the increased morbidity rates for other diseases led to ongoing recommendations from the Ministry of Health to respond to the demand for healthcare services. These recommendations included contingency plans to reinforce human resources and extend the opening hours of health centers (42, 44). These measures required a greater availability of healthcare professionals. Notably, Portugal has had a chronic shortage of nurses, with 7.5 nurses per 1000 inhabitants in 2021, one of the lowest ratios in the European Union (22). Even before the pandemic, studies indicated a shortage of nurses, primarily due to demographic aging (43). Alongside the context of the COVID-19 pandemic, demographic aging has contributed to increased demand for emergency rooms in Portugal (41).

The period of our investigation was marked by increased tension and labor conflicts in emergency rooms due to overwork during extraordinary shifts and a shortage of medical and nursing staff (43). Daily media coverage showcased this tension, with hospital emergency departments experiencing unprecedented patient influx, particularly in the Lisbon area. The combination of increased morbidity and mortality rates, rising demand for emergency rooms, cumulative fatigue among nurses due to 3 years of the COVID-19 pandemic, staff shortages, and a tense work climate all contribute to understanding the high burnout rates observed in our study.

### 4.1 Limitations and future directions

This study covered a broad region corresponding to the Lisbon metropolitan area, the most populous in the country. The study's objectives focused on an analysis carried out in a post-COVID-19 period. Even though the most severe phase of the pandemic was already passed, the results reflected a domain that has yet to be studied, as it is still very recent, and which concerns the consequences of the pandemic on health systems. The pertinence is clear when revealing the persistence of high levels of burnout in nurses who work in emergency rooms. However, it is essential to acknowledge this study's limitations and consider future research directions. Firstly, the limited size and composition of the sample. The nursing profession is predominantly female, and the sample may have needed to represent the proportion of male nurses adequately. Future studies should include a larger and more diverse sample of nurses to obtain a more representative sample, considering sex balance. Secondly, this study focused solely on the metropolitan area of Lisbon, which represents urban and densely populated areas. The findings may not be generalizable to nurses working in rural or other non-urban settings, especially considering other literature has described these settings specifically (45–47). Thirdly, it is essential to recognize that the context of instability and dissatisfaction with working conditions may have influenced the responses obtained in self-report surveys. This could lead to an overestimation of the reported levels of burnout. Future studies could incorporate multiple data collection methods, such as interviews or observational measures, to complement self-report surveys and provide a more comprehensive assessment of burnout levels. Finally, given the relatively recent nature of the COVID-19 pandemic, continuous research is necessary to monitor healthcare professionals' mental health and overall wellbeing. Longitudinal studies that follow nurses over time, considering the long-term impact of the pandemic on burnout and related factors, would provide valuable insights into the evolving nature of burnout in the aftermath of the COVID-19 pandemic.

## 5 Conclusion

This study's results unveil alarming burnout levels among nurses working in emergency rooms. These findings are particularly concerning as the data collection took place in a post-COVID-19 timeframe. However, it is crucial to analyze this distressing assessment within the broader context experienced by nurses during the post-COVID rebound. With 3 years having elapsed since the onset of the COVID-19 pandemic, the study demonstrates the enduring effects of the crisis, which have resulted in an escalated demand for healthcare services.

The findings indicate that certain factors contribute to a lower risk of severe burnout. Specifically, conditions of safety and stability in the workplace, personal investment in postgraduate training through specialization in nursing, and work experience emerged as protective factors in this study.

These findings highlight the importance of creating safe and stable work environments for nurses, supporting their professional development through postgraduate training and specialization, and recognizing the value of work experience. By addressing these factors, healthcare organizations can help mitigate the risk of severe burnout among nurses in the post-COVID-19 period and promote their overall wellbeing and job satisfaction.

## Data Availability

The original contributions presented in the study are included in the article/supplementary material, further inquiries can be directed to the corresponding author.

## References

[1] Freudenberger HJ. Staff burn-out. J Soc Issues. (1974) 30:159–65. doi: 10.1111/j.1540-4560.1974.tb00706.x

[2] Maslach C Jackson S. The measurement of experienced burnout. J Organ Behav. (1981) 2:99–113. doi: 10.1002/job.4030020205

[3] Harrison JE Weber S Jakob R Chute CG. ICD-11: an international classification of diseases for the twenty-first century. BMC Med Inform Decis Mak. (2021) 21:206. doi: 10.1186/s12911-021-01534-6 34753471 PMC8577172

[4] World Health Organization. International Classification of Diseases 11th Revision (2022). Available online at: https://icd.who.int/ct11/icd11_mms/en/release (Accessed May 18, 2023).

[5] Civita M Laurita E Di Stefano C Gervasoni M Viotti S Zucchi S. Physicians and nurses' burnout in the emergency departments of North West of Italy. Intern Emerg Med. (2021) 16:1381–5. doi: 10.1007/s11739-020-02577-9 33387199

[6] Woo T Ho R Tang A Tam W. Global prevalence of burnout symptoms among nurses: a systematic review and meta-analysis. J Psychiatr Res. (2020) 123:9–20. doi: 10.1016/j.jpsychires.2019.12.015 32007680

[7] Adriaenssens J de Gucht V Maes S. The impact of traumatic events on emergency room nurses: findings from a questionnaire survey. Int J Nurs Stud. (2012) 49:1411–22. doi: 10.1016/j.ijnurstu.2012.07.003 22871313

[8] Pei J Wang X Chen H Zhang H Nan R Zhang J . Alexithymia, social support, depression, and burnout among emergency nurses in China: a structural equation model analysis. BMC Nurs. (2021) 20:194. doi: 10.1186/s12912-021-00702-3 34629068 PMC8503998

[9] Martins V Serrão C Teixeira A Castro L Duarte I. The mediating role of life satisfaction in the relationship between depression, anxiety, stress and burnout among Portuguese nurses during COVID-19 pandemic. BMC Nurs. (2022) 21:188. doi: 10.1186/s12912-022-00958-3 35850892 PMC9289090

[10] Yuan Y Wang Z Shao Y Xu X Lu F Xie F . Dispositional mindfulness and post-traumatic stress symptoms in emergency nurses: multiple mediating roles of coping styles and emotional exhaustion. Front Psychol. (2022) 13:787100. doi: 10.3389/fpsyg.2022.787100 35391967 PMC8982862

[11] Rees CS Heritage B Osseiran-Moisson R Chamberlain D Cusack L Anderson J . Can we predict burnout among student nurses? An exploration of the ICWR-1 model of individual psychological resilience. Front Psychol. (2016) 7:1072. doi: 10.3389/fpsyg.2016.01072 27486419 PMC4949488

[12] Zhang YR Hu RF Liang TY Chen JB Wei Y Xing YH . Applying formative evaluation in the mentoring of student intern nurses in an emergency department. Front Public Health. (2022) 10:974281. doi: 10.3389/fpubh.2022.974281 36339220 PMC9626992

[13] Alqahtani AM Awadalla NJ Alsaleem SA Alsamghan AS Alsaleem MA. Burnout syndrome among emergency physicians and nurses in Abha and Khamis Mushait Cities, Aseer Region, Southwestern Saudi Arabia. ScientificWorldJournal. (2019) 2019:4515972. doi: 10.1155/2019/4515972 30906233 PMC6398028

[14] Sahraian A Fazelzadeh A Mehdizadeh AR Toobaee SH. Burnout in hospital nurses: a comparison of internal, surgery, psychiatry and burns wards. Int Nurs Rev. (2008) 55:62–7. doi: 10.1111/j.1466-7657.2007.00582.x 18275537

[15] Piotrowska A Lisowska A Twardak I Włostowska K Uchmanowicz I Mess E . Determinants affecting the rationing of nursing care and professional burnout among oncology nurses. Int J Environ Res Public Health. (2022) 19:7180. doi: 10.3390/ijerph19127180 35742428 PMC9222562

[16] Wójcik G Wontorczyk A Barańska I. Job demands, resources and burnout among polish nurses during the late wave of COVID-19 pandemic: the mediating role of emotional labor. Front Psychiatry. (2022) 13:931391. doi: 10.3389/fpsyt.2022.931391 35898625 PMC9309251

[17] Kowalczuk K Krajewska-Kułak E Sobolewski M. Working excessively and burnout among nurses in the context of sick leaves. Front Psychol. (2020) 11:285. doi: 10.3389/fpsyg.2020.00285 32158416 PMC7052176

[18] Nimako B Basatan T. Burnout and job performance of nurses in adult care settings during COVID-19 pandemic. Int J Nurs Sci. (2022) 12:1–14. doi: 10.5923/j.nursing.20221201.01

[19] Chahbounia R Gantare A. A pilot study to assess the effect of coaching on emergency nurses' stress management. Nurs Rep. (2023) 13:179–93. doi: 10.3390/nursrep13010019 36810270 PMC9944106

[20] Bernaldo-De-Quirós M Piccini AT Gómez MM Cerdeira JC. Psychological consequences of aggression in pre-hospital emergency care: cross sectional survey. Int J Nurs Stud. (2015) 52:260–70. doi: 10.1016/j.ijnurstu.2014.05.011 24947754

[21] Gillespie GL Gates DM Berry P. Stressful incidents of physical violence against emergency nurses. Online J Issues Nurs. (2013) 18:2. doi: 10.3912/OJIN.Vol18No01Man02 23452198

[22] Pordata. Increase in Emergency Room Admissions (2023). Available online at: https://www.pordata.pt/db/portugal/ambiente+de+consulta/tabela (accessed June 23, 2023).

[23] Canadian Institute for Health Information. NACRS Emergency Department Visits and Lengths of Stay (2023). Available online at: https://www.cihi.ca/en/nacrs-emergency-department-visits-and-lengths-of-stay (Accessed October 28, 2025).

[24] Moscu CA Marina V Dragomir L Anghele AD Anghele M. The impact of burnout syndrome on job satisfaction among emergency department nurses of emergency clinical county hospital “Sfântul Apostol Andrei” of Galati, Romania. Medicina. (2022) 58:1516. doi: 10.3390/medicina58111516 36363475 PMC9698052

[25] Meneguin S Ignácio I Pollo CF Honório HM Patini MSG de Oliveira C . Burnout and quality of life in nursing staff during the COVID-19 pandemic. BMC Nurs. (2023) 22:14. doi: 10.1186/s12912-022-01168-7 36635750 PMC9834673

[26] Yuguero O Rius N Soler-González J Esquerda M. Increase of burnout among emergency department professionals due to emotional exhaustion during the SARS-Cov2 pandemic: Evolution from 2016 to 2021. Medicine. (2022) 101:e31887. doi: 10.1097/MD.0000000000031887 36451498 PMC9704866

[27] Galanis P Vraka I Fragkou D Bilali A Kaitelidou D. Nurses' burnout and associated risk factors during the COVID-19 pandemic: a systematic review and meta-analysis. J Adv Nurs. (2021) 77:3286–302. doi: 10.1111/jan.14839 33764561 PMC8250618

[28] Dall'Ora C Ball J Reinius M Griffiths P. Burnout in nursing: a theoretical review. Human Resources for Health. (2020) 18:41. doi: 10.1186/s12960-020-00469-9 32503559 PMC7273381

[29] Maslach C Jackson S Leiter M. The Maslach Burnout Inventory Manual (3rd Edn.). Palo Alto, CA: Consulting Psychologists Press (1996). p. 191–218.

[30] Vicente CS Oliveira RA Maroco J. Análise Fatorial do Inventário de Burnout de Maslach (MBI-HSS) em profissionais portugueses. Psicologia, Saúde e Doenças. (2013) 14:152–67.

[31] Marôco J Marôco AL Leite E Bastos C Vazão MJ Campos J . Burnout em Profissionais da Saúde Portugueses: Uma Análise a Nível Nacional [Burnout in Portuguese Healthcare Professionals: An Analysis at the National Level]. Acta Med Port. (2016) 29:24–30. Portuguese. doi: 10.20344/amp.6460 26926895

[32] Elmore LC Jeffe DB Jin L Awad MM Turnbull IR. National survey of burnout among US general surgery residents. J Am Coll Surg. (2016) 223:440–51. doi: 10.1016/j.jamcollsurg.2016.05.014 27238875 PMC5476455

[33] Bogiatzaki V Frengidou E Savakis E Trigoni M Galanis P Anagnostopoulos F . Empathy and burnout of healthcare professionals in public hospitals of Greece. Int J Caring Sci. (2019) 12:611–26.

[34] Castro C Antunes R Fernandes JB Reisinho J Rodrigues R Sardinha J . Perceptions and representations of senior nursing students about the transition to professional life during the COVID-19 pandemic. Int J Environ Res Public Health. (2022) 19:4466. doi: 10.3390/ijerph19084466 35457334 PMC9027933

[35] Fernandes JB Vareta D Fernandes S Almeida AS Peças D Ferreira N . Rehabilitation workforce challenges to implement person-centered care. Int J Environ Res Public Health. (2022) 19:3199. doi: 10.3390/ijerph19063199 35328886 PMC8950126

[36] Zhang H Xiao Y Dai T Li Q Huang L Huang X . A cross-sectional study on burnout and its individual and environmental correlates among hepatological surgery nurses in Hunan Province, China. PLoS ONE. (2023) 18:e0283373. doi: 10.1371/journal.pone.0283373 36952501 PMC10035911

[37] Tomaszewska K Majchrowicz B Snarska K Guzak B. Psychosocial burden and quality of life of surveyed nurses during the SARS-CoV-2 pandemic. Int J Environ Res Public Health. (2023) 20:994. doi: 10.3390/ijerph20020994 36673750 PMC9859002

[38] Bartosiewicz A Januszewicz P. Readiness of polish nurses for prescribing and the level of professional burnout. Int J Environ Res Public Health. (2018) 16:35. doi: 10.3390/ijerph16010035 30586884 PMC6339262

[39] Kashgsaray N Soleimanpour M Behmanesh S Soleimanpour H. Burnout in healthcare professionals during the COVID-19 crisis. Arch Anesthesiol Critical Care. (2024) 11:3–8. doi: 10.18502/aacc.v11i1.17484

[40] Health regulatory Authority. Impact of the COVID-19 Pandemic on the Health System—March to June 2020 (2020). New Delhi: Health regulatory Authority.

[41] Organization for Economic Co-operation and Development. Portugal: Country Health Profile 2021, State of Health in the EU. European Observatory on Health Systems and Policies (2021). Paris: OECD Publishing

[42] DGS/Ministry of Health. Directorate-General for Health. Seasonal Health Response Report - Surveillance and Monitoring. Report no. 2 (2022). New Delhi: Ministry of Health.

[43] Silva S Torres A Nunes B Rodrigues A. Mortality monitoring 2022 (2023). Lisbon: Instituto Nacional de Saúde Doutor Ricardo Jorge.

[44] DGS/Ministry of Health. Directorate-General for Health. Seasonal Health Response Report - Surveillance and Monitoring. Report no. 15 (2023). New Delhi: Ministry of Health.

[45] Malik M Penalosa M Busch IM Burhanullah H Weston C Weeks K . Rural healthcare workers' well-being: a systematic review of support interventions. Families Syst Health. (2024) 42:355. doi: 10.1037/fsh0000921 39418422 PMC12079686

[46] Chunta K Robb M Hoffman R Gerwick M Zuraikat N. Examining psychological well-being and predictors of burnout in Registered Nurses (RNs) employed in rural acute care settings. Hosp Top. (2024) 1–6. doi: 10.1080/00185868.2024.2422120 39474784

[47] Holland C Malatzky C Pardosi J. What do nurses practising in rural, remote and isolated locations consider important for attraction and retention?: A scoping review. Rural Remote Health. (2024) 24:1–13. doi: 10.22605/RRH8696 39307544

